# Establishing and characterizing patient-derived xenografts using pre-chemotherapy percutaneous biopsy and post-chemotherapy surgical samples from a prospective neoadjuvant breast cancer study

**DOI:** 10.1186/s13058-017-0920-8

**Published:** 2017-12-06

**Authors:** Jia Yu, Bo Qin, Ann M. Moyer, Jason P. Sinnwell, Kevin J. Thompson, John A. Copland, Laura A. Marlow, James L. Miller, Ping Yin, Bowen Gao, Katherine Minter-Dykhouse, Xiaojia Tang, Sarah A. McLaughlin, Alvaro Moreno-Aspitia, Anthony Schweitzer, Yan Lu, Jason Hubbard, Donald W. Northfelt, Richard J. Gray, Katie Hunt, Amy L. Conners, Vera J. Suman, Krishna R. Kalari, James N. Ingle, Zhenkun Lou, Daniel W. Visscher, Richard Weinshilboum, Judy C. Boughey, Matthew P. Goetz, Liewei Wang

**Affiliations:** 1 Department of Molecular Pharmacology and Experimental Therapeutics, Mayo Clinic, 200 First Street SW, Rochester, MN 55905 USA; 2 Department of Oncology, Mayo Clinic, Rochester, MN 55905 USA; 3 Department of Laboratory Medicine and Pathology, Mayo Clinic, Rochester, MN 55905 USA; 4 Department of Health Sciences Research, Mayo Clinic, Rochester, MN 55905 USA; 5 Department of Cancer Biology, Mayo Clinic, Jacksonville, FL 32224 USA; 6 Department of Surgery, Mayo Clinic, Jacksonville, FL 32224 USA; 7 Department of Hematology/Oncology, Mayo Clinic, Jacksonville, FL 32224 USA; 8 Affymetrix, now part of Thermo Fisher Scientific, Santa Clara, CA 95051 USA; 9 Department of Hematology/Oncology, Mayo Clinic, Scottsdale, AZ 85259 USA; 10 Department of Surgery, Mayo Clinic, Scottsdale, AZ 85259 USA; 11 Department of Radiology, Mayo Clinic, Rochester, MN 55905 USA; 12 Department of Surgery, Mayo Clinic, Rochester, MN 55905 USA; 13 Department of Surgery, Cedars-Sinai Medical Center, Los Angeles, CA 90048 USA

**Keywords:** Breast cancer, Patient-derived Xenograft (PDX), Percutaneous tumor biopsies (PTB), Prospective neoadjuvant chemotherapy (NAC), Pre-clinical therapy

## Abstract

**Background:**

Patient-derived xenografts (PDXs) are increasingly used in cancer research as a tool to inform cancer biology and drug response. Most available breast cancer PDXs have been generated in the metastatic setting. However, in the setting of operable breast cancer, PDX models both sensitive and resistant to chemotherapy are needed for drug development and prospective data are lacking regarding the clinical and molecular characteristics associated with PDX take rate in this setting.

**Methods:**

The *Breast Cancer Genome Gu*ided *T*herapy Stud*y* (BEAUTY) is a prospective neoadjuvant chemotherapy (NAC) trial of stage I-III breast cancer patients treated with neoadjuvant weekly taxane+/-trastuzumab followed by anthracycline-based chemotherapy. Using percutaneous tumor biopsies (PTB), we established and characterized PDXs from both primary (untreated) and residual (treated) tumors. Tumor take rate was defined as percent of patients with the development of at least one stably transplantable (passed at least for four generations) xenograft that was pathologically confirmed as breast cancer.

**Results:**

Baseline PTB samples from 113 women were implanted with an overall take rate of 27.4% (31/113). By clinical subtype, the take rate was 51.3% (20/39) in triple negative (TN) breast cancer, 26.5% (9/34) in HER2+, 5.0% (2/40) in luminal B and 0% (0/3) in luminal A. The take rate for those with pCR did not differ from those with residual disease in TN (*p* = 0.999) and HER2+ (*p* = 0.2401) tumors. The xenografts from 28 of these 31 patients were such that at least one of the xenografts generated had the same molecular subtype as the patient. Among the 35 patients with residual tumor after NAC adequate for implantation, the take rate was 17.1%. PDX response to paclitaxel mirrored the patients’ clinical response in all eight PDX tested.

**Conclusions:**

The generation of PDX models both sensitive and resistant to standard NAC is feasible and these models exhibit similar biological and drug response characteristics as the patients’ primary tumors. Taken together, these models may be useful for biomarker discovery and future drug development.

**Electronic supplementary material:**

The online version of this article (doi:10.1186/s13058-017-0920-8) contains supplementary material, which is available to authorized users.

## Background

Like many cancers, breast cancer is a heterogeneous disease [1]. Clinical subtypes for breast cancer are based on the immunohistochemical (IHC) determination of estrogen receptor (ER), progesterone receptor (PR) and human epidermal growth factor receptor 2 (HER2) [2, 3]. Breast cancer can be classified into five subtypes based on clinical and IHC criteria, namely, triple negative (ER-/PR-/HER2-), ER+/HER2+, ER-/HER2+, luminal A and luminal B subtypes which differ in clinical outcomes and optimal treatment strategies [4]. Multimodality therapies which target ER and HER2 as well as polychemotherapy have significantly improved clinical outcomes [5–7]. However, treatment responses are inconsistent, likely due in part to the complexity of the tumor and host genomes [6, 8, 9]. A deeper understanding of the mechanisms involved in drug response is necessary to achieve the goal of individualized drug therapy. This knowledge would also guide future drug development and help make it possible to overcome resistance to “standard” chemotherapy [8, 10].

One challenge associated with effectively identifying and understanding the mechanisms of drug resistance is the availability of in vivo models that can faithfully represent human tumor biology and have a high possibility of translating finding with the models to patients. Conventional xenograft models generated with cancer cell lines have limitations, such as inability to capture tumor heterogeneity [1, 11, 12] or recapitulate the spectrum of human breast cancer due to distinct differences in mice strains, cell origins and the tumor microenvironment, all of which limit their predictive value for clinical application [13, 14].

Recent studies have shown that xenografts developed from patient tumors, commonly referred to as patient-derived xenografts (PDX), may better recapitulate the molecular complexity and heterogeneity of a human tumor [15]. They have also been shown to maintain the cell morphology, architecture, microenvironment and molecular signatures of the original patient tumors [16, 17]. Evidence suggests that PDX models come closer to simulating human cancer than do cell lines, and the National Cancer Institute (NCI) is developing PDX models as a potential substitute for the NCI-60 cell lines [18]. PDX models have also become an effective tool for testing drugs to help accelerate the translation of research from bench to bedside [19–21]. Although PDXs have been used extensively with pancreatic cancer, liver cancer and brain tumors with high take rates [22], breast cancer PDX rates of stable transplantation have been less successful, with take rates of 20% or less [23, 24]. Perhaps most importantly, the clinical outcomes of patients from whom these xenografts were derived are usually unknown [19, 25], which makes our models unique in this aspect.

Here we report detailed analysis of PDXs generated from the prospective Breast Cancer Genome Guided Therapy Study (BEAUTY). In the BEAUTY study, serial percutaneous tumor biopsy (PTB) and surgical samples were obtained for the generation of PDXs prior to and following neoadjuvant chemotherapy (NAC) in women with high-risk primary breast cancer (the study design is shown in Additional file 1: Figure S1) [26]. We sought to evaluate the feasibility of establishing PDXs from percutaneous tumor biopsies and from chemotherapy resistant tumor at surgery and to assess the factors influencing PDX take rate. Additionally, we sought to compare the histologic and molecular profiles of the PDXs with the clinical outcomes of the patients (chemotherapy resistant versus chemotherapy sensitive) from whom the PDXs were derived. Finally, given prior reports that “take rate” is associated with a worse clinical outcome in the metastatic setting [27], we sought to determine if take rate was associated with chemotherapy response (pCR) which is a determinant of survival in neoadjuvant-treated breast cancer.

## Methods

### Patient tissue acquisition

Patients with newly diagnosed stage I to Ш breast cancer who were recommended for NAC at Mayo Clinic were eligible for study participation. The protocol was approved by the Mayo Clinic Institutional Review Board (IRB). All patients who participated in this study provided written informed consent. Ultrasound-guided baseline percutaneous core-needle biopsies of the primary breast tumor were obtained after study enrollment at Mayo Clinic Rochester (MCR) or Mayo Clinic Florida (MCF). Fourteen-gram spring-loaded core needle devices were used. After finishing standard neoadjuvant weekly taxane +/- trastuzumab followed by anthracycline-based chemotherapy, samples of residual disease from surgical resection were also obtained whenever possible. Fresh tumor tissue was obtained and a portion of the fresh tumor tissue was kept on ice in sterile phosphate-buffered saline (PBS, Thermo Fisher Scientific, Waltham, MA, USA) for implantation in NOD-SCID (NOD.CB17-*Prkdc* ^*scid*^/J) or NSG (NOD.Cg-*Prkdc* ^*scid*^ *Il2rg* ^*tm1Wjl*^/SzJ) mice within 30–60 minutes. Additional biopsy or surgical samples were frozen or placed in formalin and embedded in paraffin for later analysis. MCF and MCR used similar procedures to process and transplant tissue fragments.

### Establishment of patient-derived subcutaneous xenografts

The Mayo Clinic Institutional Animal Care and Use Committee reviewed and approved all of the mouse experiments. Six- to 8-week-old female mice were maintained and pretreated with 17β-estradiol as described in the Additional file 2: Supplementary Methods. Baseline pre-treatment percutaneous biopsy specimen and post-treatment surgical samples were delivered in sterile PBS and were received within 1 hour of the biopsy/surgery. Samples were implanted subcutaneously and tumors were monitored on a daily basis. When xenograft primary tumors reached approximately 200–1500 mm^3^, mice were sacrificed and tissue fragments were transplanted to new mice to expand the xenograft tissue. At each passage, whenever possible, tumor samples were also fixed in formalin for histology and flash frozen for subsequent genomic or protein analysis. Tumors were also preserved for future engraftment by freezing in liquid nitrogen in preserving solution that consisted of DMEM with 20% FBS and 10% DMSO. Tumor take rate was defined as percent of patients with the development of at least one stably transplantable (passed at least for four generations) xenograft that was pathologically confirmed as human breast cancer [23].

### Histologic evaluation of patient tumor and corresponding xenograft tumors

The morphology and immunohistochemical staining pattern for both primary tumors and corresponding xenograft tumors were evaluated. All tumor samples were fixed within 1 hour of resection in 10% neutral buffered formalin for 6–72 hours, followed by paraffin embedding, according to guidelines. In addition to routine hematoxylin and eosin (H&E) staining, all tumors were evaluated by IHC staining for ER, PR, HER2, and Ki-67. Fluorescence in situ hybridization (FISH) assay was also performed whenever necessary for determination of HER2 amplification. Antibodies and detailed staining methods are described in the Additional file 2: Supplementary Methods. Patient tumors were stained in the Mayo Clinic Immunostains Laboratory using standard clinical protocols, while the xenograft tumors were stained in the Mayo Clinic Pathology Research Core. The clinical approximated subtypes of breast cancer were defined according to the 2011 St Gallen International Breast Cancer Conference as: luminal A (with ER >10% + tumor grade 1 or ER >10% + tumor grade 2 + Ki-67 0-14%); luminal B (ER >10% + tumor grade 2 + Ki-67 > 14% or ER >10% + tumor grade 3); ER+/HER2+ (ER >10% + HER2+ (3+ by IHC or amplified by FISH); ER-/HER2+ (ER ≤10% + HER2+ (3+ by IHC or amplified by FISH); and triple negative (TN) (ER ≤10% + any PR + HER2-).

### Microarray and subtype analysis

Ninety-four pathologically confirmed xenograft samples from baseline tumors together with 11 replicates corresponding to 23 unique patients were processed and analyzed using the Affymetrix HTA2.0 Array (GeneChip® Human Transcriptome Array 2.0). Rather than using all probes available on the microarray for transcriptome profiling, a unique probe filtering process was applied. Here, the best effort was made to exclude from analysis those probes possessing significant homology with the murine genome and transcriptome in order to interrogate human specific signals of the PDXs. Further details on the probe selection, procedures of extraction and qualification of xenograft tumor mRNA are described in the Additional file 2: Supplementary Methods. Affymetrix gene expression array analyses were performed in the Medical Genome Facility Gene Expression Core at Mayo Clinic Rochester according to standard protocols recommended by Affymetrix. Standard QC analysis workflows were applied using the Affymetrix Expression Console Software v1.4.1.46 [28].

### Xenograft treatment response

Tumors were implanted and grown in NOD-SCID or NSG mice. Once xenograft tumors grew to 1 cm in diameter, mice were sacrificed and tumors were reimplanted in additional NOD-SCID mice. Tumor growth was monitored twice weekly. For drug treatment experiments, once tumors reached 150–250 mm^3^, mice were randomized into control (vehicle, castor oil 1:10 v/v) or paclitaxel (Sigma-Aldrich, St. Louis, MO, USA; Cat.No.T7402, 20 mg/kg, i.p. once every 3 days) groups, with each group consisting of 7 to 8 mice.

### Statistical analysis and data visualizations

Fisher’s exact test was used to assess whether tumor take rate differed with respect to disease characteristics of the patient or type of mouse implanted. Correlation coefficients were calculated using the Spearman rank formula. Tumor growth curves were plotted using GraphPad Prism 5 software (GraphPad Software, San Diego, CA, USA). Student *t* test was used to compare continuous variables. *P* value of less than 0.05 was considered statistically significant. Visualizations were generated using the R packages: beanplot v1.2 [29], Heatplus v2.16.0 [30] and ape v3.3 [31].

## Results

### Establishment of patient-derived xenografts

A total of 140 patients were enrolled in the BEAUTY study between March 2012 and May 2014. Pre-neoadjuvant percutaneous biopsy samples from 120 patients with adequate sample were implanted subcutaneously in 412 immunodeficient mice. There was tumor growth among the tissues implanted from seven of these 120 patients but tissue was not available for pathologic confirmation of human breast cancer and as a result, tissue from these patients were excluded from further analyses. Of the remaining 113 patients, 54 tumor samples implanted from 38 patients had tumor growth. Pathological assessment of these PDXs found that 12 PDXs growth corresponding to seven patients did not include any human-breast cancer. The tumors growing were found to be human or murine lymphoma (n = 5), murine mammary tumor (n = 3), murine osteosarcoma (n = 2), and murine hemangiosarcoma (n = 2). Thus, the PDX take rate for these percutaneous pre-treatment biopsies was 27.4% (31/113; 95%CI: 19.5–36.6%). There were no PDXs established in the nine luminal A and 1 luminal unknown (unable to establish whether luminal A or luminal B) tumors. The take rate was 51.3% (21/39; 95% CI: 34.8-67.6%) in TN subtype; 26.5% (9/34; 95% CI: 12.9–44.4%) in the HER2+ subtype; and 6.7% (2/30; 95% CI: 0.1–22.1%) in the luminal B subtype (Table 1).

**Table 1:** Pre-treatment biopsy PDX by clinical molecular subtype

Clinical molecular subtype	Total implanted^a^	Any tumor growth	Verified human breast tumor (%)	pCR	
				no	yes
ER-/HER2+	20	6	5 (25.0)	4/8	1/12
ER+/HER2+	14	5	4 (28.6)	3/11	1/3
LumA	9	0	0 (0.0)	0/9	0/0
LumB	30	6	2 (6.7)	0/27	2/3
LumUnk	1	0	0 (0.0)	0/1	0/0
Triple negative	39	21	20 (51.3)	9/17	11/22
Total	113	38	31 (27.4)	16/73	15/40

*Abbreviations*: *PDX* patient-derived xenograft, *pCR* pathological complete response, *ER* estrogen receptor, *HER2* human epidermal growth factor receptor 2, *LumA* luminal A, *LumB* luminal B, *LumUnk* luminal unknown

^a^An additional seven tumors were implanted and had growth but were not available for pathological confirmation n

In addition, surgical residual tumor samples post chemotherapy obtained from 35 patients were implanted into 184 mice (on average five mice per patient). Tumor growth pathologically confirmed to be human breast cancer was seen in the surgical residual tumor samples from six patients. Thus, the take rate for the post-chemotherapy residual tumors was 17.1% (6/35; 95% CI: 6.6–33.7%) (Table 2). There was no take among the three luminal A and one luminal unknown tumors. However, four of the nine TN, two of the 18 Luminal B, and one of the eight HER2+ post-chemotherapy residual tumors took.

**Table 2:** Surgical sample PDX by clinical molecular subtype

Clinical molecular subtype	Total implanted	Any tumor growth	Verified breast tumor (%)
ER-/HER2+	3	1	1 (33.3)
ER+/HER2+	5	0	0 (0.0)
LumA	3	0	0 (0.0)
LumB	18	2	0 (0.0)
LumUnk	1	0	0 (0.0)
Triple negative	9	4^b^	5 (55.6)
Totals	35^a^	7	6 (17.1)

Abbreviations: *PDX* patient-derived xenograft, *ER* estrogen receptor, *HER2* human epidermal growth factor receptor 2, *LumA* luminal A, *LumB* luminal B, *LumUnk* luminal unknown

^a^An additional two tumors were implanted and had growth but were not available for pathological confirmation

^b^One triple negative PDX did not grow to the defined growth threshold due to mouse health condition, but did passage with verification

### Association between disease and mouse characteristics and xenograft take rate

Next, we examined whether PDX tumor take rates differed with respect to patient clinical parameters (ER, HER2, and grade) or host mice strains. In the pre-neoadjuvant biopsy tissue, take rate was found to differ significantly by clinical molecular subtype (extended Fisher’s exact test *p* < 0.001) (Table 1). There were only two PDXs generated from the 40 patients with luminal breast cancers (Table 1). Among 34 HER2+ and 39 TN breast cancers (Additional file 2: Table S1), univariately, the take rate was found to be: greater in grade 3 tumors than grade 1–2 tumors (*p* = 0.0003; difference = 46.6%; 95% CI: 29.9–63.3%); greater in NSG mice than in NOD-SCID mice (*p* = 0.0012; difference = 39.9%; 95% CI: 17.7–62.0%); and greater in TN tumors than HER2+ tumors (*p* = 0.0348; difference = 24.8%; 95% CI: 3.2–46.4%).

### Association between patient response to chemotherapy and xenograft take rate

Of the pre-NAC PTB samples that were implanted from 113 women, PDX take rate was 27.4% (95%CI: 19.5–36.6%) (Table 1). The PDX take rate was not found to differ with respect to whether the patient had or did not have residual disease after NAC in both those with TN tumors (9/17 vs. 11/22; *p* = 0.999) and those with HER2+ tumors (7/17 vs. 2/15; p = 0.2401).

We also examined whether PDX tumor take rates differed for the residual surgical samples. There were no PDXs generated from residual surgical tissue of the 22 patients with luminal breast cancers. As shown in Additional file 2: Table S2, among HER2+ and TN residual breast cancers, the take rate was found univariately to be greater in residual tumors with Ki-67 > 14% than the residual tumors with Ki-67 ≤ 14% (*p* = 0.0280) and somewhat greater in TN disease than HER2-enriched disease (*p* = 0.0882).

### Pathological analysis of xenograft tumors

**Fig. 1:**
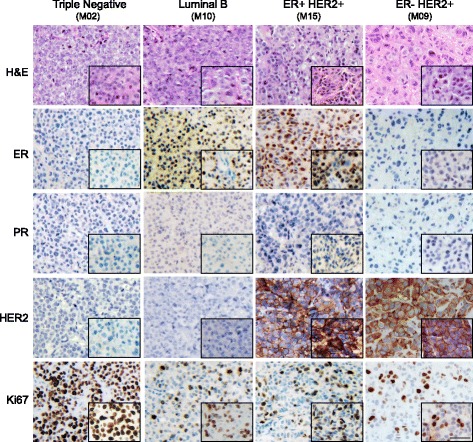
Comparisons of histological and biomarker characteristics of PDX and their corresponding original patient tumors. Four representative passage 2 PDX models with different clinical subtypes based on ER, PR, and HER2 status, and their corresponding patient tumors are shown. The histology was verified using H&E staining and the expression of ER, PR, HER2, and Ki-67 was visualized using immunohistochemistry. Xenograft tumors are shown in the *background* with their corresponding human tumors shown in the *bottom right inserts*. (Scale bar, 50 μm). *ER* estrogen receptor, *H&E* hematoxylin and eosin, *HER2* human epidermal growth factor receptor 2, *PR* progesterone receptor

**Fig. 2:**
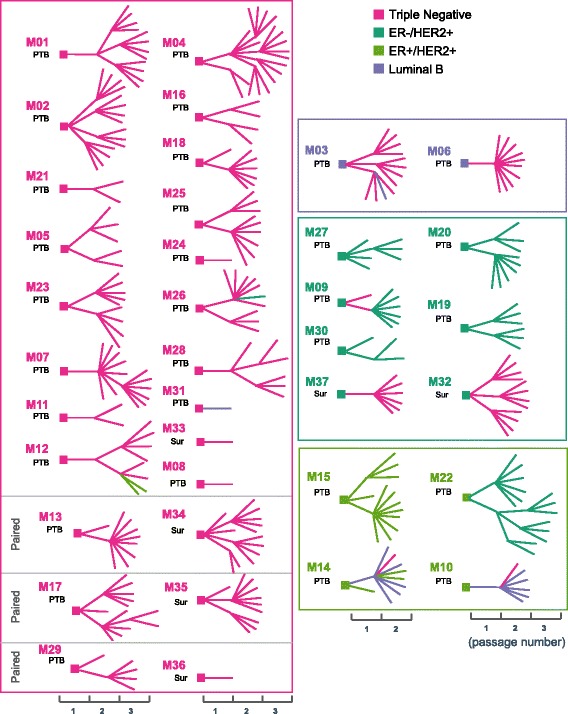
Changes in pathological subtypes within lineages for PDX models generated from both baseline percutaneous biopsy samples and surgical samples post-chemotherapy. The diagrams show the transplant history and pathological subtype of each xenograft line. *Squares* indicate the patient tumor and line segments represent different xenograft transplants. Colors represent the pathological subtypes determined by immunohistochemical staining of biomarkers (*pink*: triple negative; *dark green*: ER-/HER2+; *yellow green*: ER+/HER2+; *purple*: luminal B). The tumor origin is indicated (*PTB* percutaneous biopsy, *Sur* surgical samples after chemotherapy). Up to three passages for an individual PDX are shown. *ER* estrogen receptor, *HER2* human epidermal growth factor receptor 2

The morphology of each primary patient tumor was compared to the corresponding xenografts derived from that patient, including the primary transplants with implanted human tumors as well as second and third generations of xenografts passed from the primary transplants (Figs. 1 and 2). The morphology of xenograft tumors strongly resembled that of the corresponding patient tumors; however, some differences were observed as follows: the xenografts tended to show more solid architecture with less surrounding stroma than did the primary human tumors, and most xenografts had a higher grade with little to no tubule formation, more prominent nuclear pleomorphism and a higher rate of proliferation by both mitotic count and Ki-67 staining regardless of the grade or histology of the original human tumor.

In addition to morphology, tumor subtypes based on immunohistochemistry were also compared between patient tumors and xenografts. The xenografts from 28 of 31 patients were such that at least one of the xenografts generated had the same molecular subtype as the patient (Additional file 2: Table S3). Comparisons between representative xenografts and their corresponding primary human breast tumors for each of the four clinical subtypes by IHC are shown in Fig. 1. Figure 2 shows the changes over time in pathological subtypes within lineages for PDX models generated from both baseline percutaneous biopsy samples and surgical samples. While many xenografts retained the same clinical subtypes as their corresponding patients’ tumors, heterogeneity was noted. For example, xenografts derived from ER-positive patients tumors tended to lose ER (Fig. 2 and Additional file 1: Figure S2). In some cases, even though luminal B tumors grew as luminal B in the xenografts, the staining of ER in nuclei was considerably weaker than that observed in the patient tumor. In other examples, the primary tumor was ER+/HER2+ prior to neoadjuvant chemotherapy. However, when this tumor was implanted into multiple mice and expanded into the next generation, there were mixed subtypes among different mice including luminal B and TN (M10 in Fig. 2). Interestingly, the post-chemotherapy surgical sample from the same patient showed a loss of HER2 expression and the human tumor subtype changed to luminal B (Additional file 2: Table S4). Finally, in one case, multiple xenografts grew from the original ER+/HER2+ baseline tumor to yield PDX with three different molecular subtypes, ER+/HER2+, luminal B, and TN (Fig. 2 and Additional file 1: Figure S3).

### Intrinsic molecular subtype classification of xenograft tumors

Gene expression profiling classifies breast cancer into different molecular subtypes [4, 32]. In order to assess molecular similarities between the xenograft samples and their original human tumors, we used PAM50 classifier genes to compare transcriptomes between xenografts and their corresponding human tumors. Of the 22 patients with pre-neoadjuvant PAM50 results and xenografts generated from their pre-neoadjuvant breast sample, 18 patients (81.8%) had xenografts with the same PAM50 classification as their pre-neoadjuvant breast samples (Additional file 2: Table S5). Of the 86 xenografts generated, 13 did not have the same PAM50 classification as compared to their respective patient matched pre-neoadjuvant breast sample.

### Comparison of PDXs across different generations

In order to determine whether transplantable xenografts were stable over time at the transcriptome level, we conducted expression array analyses on a subset of 87 PDX tumor samples from up to three different mice generations derived from 23 unique patients. Using Spearman's correlation space and complete linkage, unsupervised clustering of the xenografts was performed for the 87 PDX tumor samples. Clustering the gene expression data demonstrates high similarity among xenografts derived from the same tumor source across generational passages, regardless of the xenograft’s clinical pathology. The best example of this phenomenon is shown in the fanned dendrogram for patient M14; the first passage (M141A) model of this patient was a luminal B (Fig. 4a). This mouse model had six second generation passages including one TN, two ER+/HER2+, and three luminal B. As shown in Fig. 4a, the gene expression data from first and second generation xenografts for M141A clustered together irrespective of the pathology. However, there was an additional xenograft model (M141B, an ER+/HER2+), which also clustered with M141A but did not have subsequent passages. This sample also demonstrates that xenografts share similarity with different transplantations that yield clinically different xenografts. The intra and inter-sample correlations are depicted graphically in Fig. 4b. In all tumor subtypes, the intra xenograft correlations are slightly higher than inter xenograft correlations.

### Concordance in taxane response between xenografts and corresponding patients

To determine whether PDXs exhibit chemotherapy sensitivity similar to that observed in their corresponding patients, PDX models derived from biopsy samples (prior to neoadjuvant chemotherapy) were assessed for in vivo paclitaxel response. All PDX tumors used to assess in vivo paclitaxel response showed same pathology and histology as their corresponding human tumors. Clinical responses to paclitaxel in the patients were determined by comparing pre-chemotherapy MRI and MRI after taxane therapy (Fig. 5a and c), and were classified as complete response (CR, complete disappearance of the lesion), partial response (PR, 30% or more decrease in the longest diameter of the lesion from pre-treatment size), or stable disease (SD, reduction in size of the tumor inferior than 30%) based on the Response Evaluation Criteria in Solid Tumors (RECIST) trial [33]. In our eight PDX paclitaxel treatment study, five clinical responders, including both CR and PR, showed response as measured by change in tumor size after paclitaxel treatment (Fig. 5a and Additional file 1: Figure S4). In contrast, PDXs derived from three patients with stable disease showed minimal response to paclitaxel (Fig. 5d and Additional file 1: Figure S4). Therefore, in these eight samples, there was complete concordance in paclitaxel response between the PDX models and the corresponding patients (Additional file 2: Table S6).

## Discussion

In breast cancer, as in most solid tumors, intrinsic or acquired drug resistance is a major cause of cancer-specific death [10, 34, 35], Studying the mechanism of drug resistance is greatly hindered by the lack of in vivo models that resemble human cancer biology [25, 34]. Numerous attempts have been made to generate patient-derived transplantable xenografts over the past three decades [11, 15–17, 23]. However, propagation of hormone-dependent human cancers such as breast cancer in immunodeficient mice is challenging [1, 19, 21, 36]. PDX mice generated using samples collected at different stages during chemotherapy treatment provide unique in vivo animal models to study mechanisms of drug resistance and to help with drug development.

Successful growth of xenografts has previously been achieved mainly with high-grade advanced surgical tumor samples or tumors from breast cancer metastatic sites [16, 17, 23, 37]. However, the take rate with breast cancer percutaneous biopsy samples [25], remains unknown. Our study provides specific information on PDX models generated with percutaneous tumor biopsies during a neoadjuvant study. The primary tumor percutaneous biopsy samples were obtained from 113 chemotherapy naïve patients and the surgical samples were from 35 patients with residual disease after the completion of chemotherapy (from the same cohort of patients). In our study, we generated 37 pathologically confirmed transplantable xenograft lines, including 31 from chemotherapy naïve patients and six from residual disease after completion of taxane and anthracycline-based chemotherapy (Table 1 and 2). These models provide a valuable opportunity to facilitate drug development and personalized cancer therapy in the near future.

The PDX take rate was 27.4% for the percutaneous pre-treatment biopsies and 17.1% for the post-chemotherapy residual tumor. All samples in this report were freshly obtained from biopsies and processed for implantation within 1 hour. Previous studies have used overnight shipped samples [27] for generating PDX models. We also performed a pilot study with samples from Mayo Clinic Arizona shipped to Mayo Clinic Rochester overnight and implanted into mice. We did not observe any evidence for tumor take in these cases (nine patients, data not shown).

Consistent with previous reports [17, 23], we found that for pre-treatment percutaneous needle biopsy samples, TN breast cancer tumors and HER2+ tumors had higher take rates than luminal tumors [16, 23, 38] (Table 1). As all but two of the 40 pre- and 22 post- treatment luminal tumors failed to take, we examined the factors which might impact take rate in the subset of TN and HER2+ tumors. In the pre-treatment of TN and HER2+ tumor samples, we did not find take rates differing with respect to age 50 or older (*P* > 0.05, Additional file 2: Table S1), which differed from a previous report that tumors from younger women were associated with better take rates [39]. We also did not find that take rate differed by Ki-67 for chemotherapy naïve tumors, which may be due to the fact that over 90% of these pre-treatment tumor had a Ki-67 > 14%. We did find that take rate was higher in higher grade disease (Nottingham grade 3 tumor relative to grade 1 or 2 tumors) and that NSG mice had a higher take rate than did NOD/SCID mice (Additional file 2: Table S1). Host mouse strain has been reported to affect xenograft success rate. NOD/SCID or NOD/SCID/IL2γ-receptor null (NSG) strains have been the preferred rodent strains for the generation of PDXs due to their higher engraftment rates. NOD/SCID mice lack B cell and T cell function but retain innate cellular immunity, while NSG mice are engineered on the NOD/SCID background with a complete null allele of the IL2 receptor common gamma chain which leads to a deficiency in functional NK cells [40]. Previous reports indicated that implantation in NOD/SCID or NSG yielded similar take rates [17, 23, 41]. However, in our study, we found that NSG had a better take rate than did NOD-SCID mice (*P* = 0.0012) (Additional file 2: Table S1). This might be due to the fact that the NSG strain is known to have a longer life span and that they are healthier than NOD/SCID mice [40], both of which provide an increased chance to achieve successful xenografting. However, since our study is not designed specially to test this hypothesis, this conclusion may need to be further confirmed. The take rate in the post-chemotherapy residual tissue was not found to differ with respect to mouse strain. This could be due to the small number of NSG implanted with post-chemotherapy tumors.

In breast cancer, a prior report evaluating a limited number of patients (n = 42) in the newly diagnosed and metastatic setting suggested that engraftment of tumor samples (n = 12) was associated with shorter survival across all subjects studied [27]. In this study, we found no evidence of a difference in take rate between patients who achieved a pCR (chemotherapy-sensitive disease) and those with residual disease (chemotherapy resistance) in either TN or HER2+ breast cancers. Notably, pCR is a strong surrogate for overall survival in breast cancer [42]. Further follow-up will be necessary to determine if tumor engraftment is associated with long-term clinical outcomes in newly diagnosed breast cancer.

Take rate in the post-chemotherapy residual tissue was higher in those residual tumors with Ki-67 > 14% relative to residual tumors with Ki-67 ≤ 14%. This observation concerning the impact of Ki-67 on post-chemotherapy take rates is consistent with previous findings, suggesting that proliferative capacity might be required for tumor growth in mice [43]. The post-chemotherapy PDX models will likely be a valuable resource for understanding mechanisms of resistance to this standard chemotherapy regimen among TN or HER2+ residual tumors.

When we compared the histologic, pathologic and molecular profiles between xenografts and their corresponding human tumors, we found that, similar to previous reports, in most cases, the xenografts reflected the human tumors [17, 23]. However, especially in the luminal B subtype (Fig. 2), xenografted tumors tended to lose ER expression, which may be due to tumor heterogeneity in the primary human tumor, resulting in better “take rates” in mice for the ER negative subclones. However, we cannot exclude the possibility that the loss of ER was due to the change in microenvironment. The potential tumor heterogeneity was also reflected in other situations in which the same original human tumors, when implanted in multiple mice, resulted in PDX models with different breast cancer subtypes. One such example was in the case of M14 where three different subtypes of PDX lines were generated from the same patient biopsy sample (Additional file 1: Figure S3). Interestingly, the patient tumor also showed intratumoral heterogeneity as shown by the IHC in Additional file 1: Figure S3. In another case (M10), we found that, when a baseline ER+/HER2+ tumor was implanted into multiple mice and expanded into the next generation, there were mixed subtypes including luminal B and TN among different mice. Interestingly, the post-chemotherapy surgical sample from the same patient showed a loss of HER2 expression and the subtype became luminal B (Additional file 2: Table S4). These observations might suggest that xenografts may also, to some extent, represent the natural course of tumor progression, a hypothesis supported by other studies that found genomic changes in PDX tumors [44, 45]. It should be emphasized that in our study the needle core biopsy used for pathology review was a separate core from that implanted into the mice. Therefore, the differences could also reflect sampling different portions of the tumor and tumor heterogeneity. In addition, we also observed that in 18 of the 68 mice that had tumor growth, the tumors were pathologically confirmed as non-human-breast tumors. This observation further stresses the importance of pathological assessment for every PDX model even if they had the same origin.

**Fig. 3:**
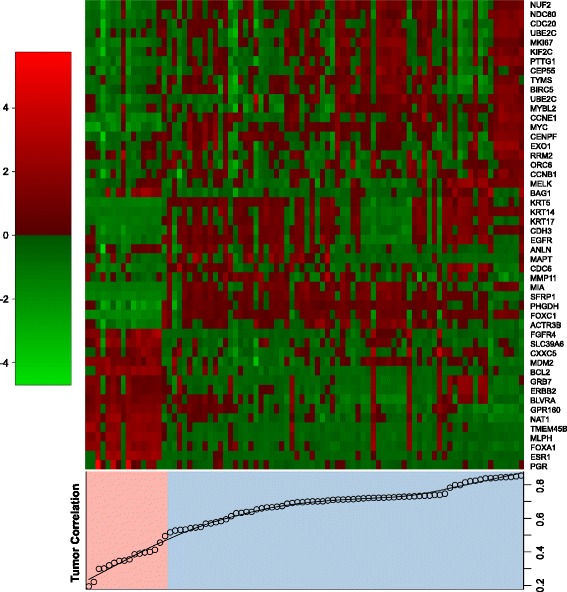
Comparisons of intrinsic signature between different xenografts and their corresponding original human tumors. The heatmap represents the intrinsic molecular subtypes based on PAM50, where the genes were clustered in one dimension using complete linkage of Spearman's correlations. When comparing PAM50 gene profiling with the original tumor source, the xenograft tumor samples were split into low (correlation below 0.5, in absolute terms, *shaded in pink*) and high correlations (above 0.5, *shaded in blue*) groups

**Fig. 4:**
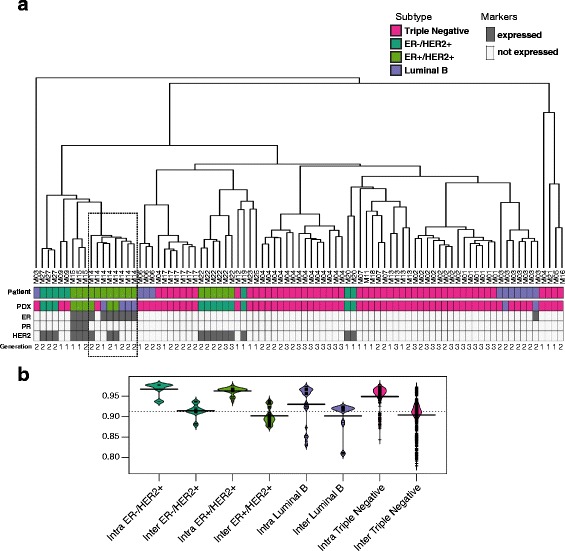
Unsupervised clustering reveals similarity among different xenograft tumors. Xenograft lines are stable over multiple transplant generations with respect to gene expression by Affymetrix human transcriptome array. **a** Unsupervised hierarchical clustering was performed based on gene expression for 87 xenografts derived from 23 unique patients. The dendrogram legend provides individual PDX lines. Xenograft tumors derived from the same patient, regardless of the number of passages, clustered more tightly together than those derived from different patient tumors. The colors indicate different clinical pathological subtypes: *pink* = TN, *dark green* = ER-/HER2+, *yellow/green* = ER+/HER2+, *purple* = luminal B. “Primary” indicates the subtype of tumor of origin. “PDX” indicates the subtype of individual PDX tumor. Passage number is indicated as *black/gray* color key. ER, PR, HER2 expression are indicated as expressed (*grey*) and not expressed (*white*). **b** The graph depicts intra and inter sample correlation among multiple generations of xenografts derived from 14 individual tumors from which multiple generations of tumors were available. *ER* estrogen receptor, *HER2* human epidermal growth factor receptor 2, *PDX* patient-derived xenograft, *PR* progesterone receptor

Gene expression profiling showed that the intrinsic breast cancer phenotypes of the xenografts were well represented and in concordance with those of the original tumors (Fig. 3), an observation that was consistent with previous reports by several other groups [46, 47]. We also observed a consistent phenotype among xenografts derived from the same patient across different generations, as shown graphically in Fig. 4a. Even though in several cases, different pathological subtypes were observed in mice, tumors derived from the same patients still clustered together based on expression array data than those derived from different patients.

**Fig. 5:**
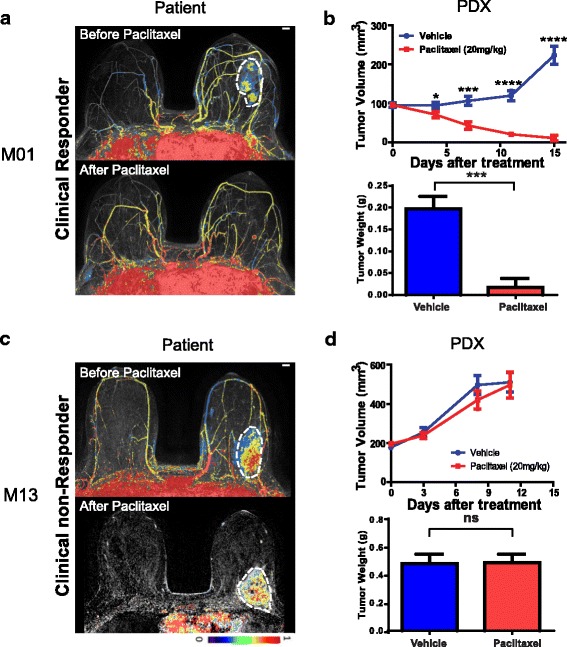
Representative PDX in vivo paclitaxel response. **a**, **c** Clinical response was assessed using radiological imaging. Representative MR imaging results before and after paclitaxel chemotherapy for a paclitaxel clinical responder (patient M01) and a clinical non-responder (patient M13) were shown. **b**, **d** Passage 4 tumors were used for the drug tests. Tumor fragments (4 mm^3^) were transplanted subcutaneously into NOD-SCID mice. Once tumors reached 150–200 mm^3^, mice were randomized to two groups (n = 6–8/group). Paclitaxel (20 mg/kg) or vehicle was administered i.p. every 3 days for 2 weeks. Tumor size and mice body weight were measured every 3–4 days. Data represents as the mean volume of xenograft tumors ± SEM. Statistical difference was analyzed by Student’s *t* test*.* *P > 0.05, **P < 0.01, ***P < 0.001, ****P < 0.0001. Tumor pictures were taken when experiments were terminated and tumor mass was quantified. *PDX* patient-derived xenograft

Finally, all eight PDX models that we tested for paclitaxel response showed perfect concordance with the clinical response of the patient from whom the biopsy had been obtained (Fig. 5b, 5d, Additional file 1: Figure S4 and Additional file 2: Table S6).

PDXs provide biologically relevant in vivo models for drug screening. Recently, Gao and Sellers [48] demonstrated both the reproducibility and the clinical translatability of the use of PDX models for screening compounds and for assessing the potential of clinical therapeutic modalities. Our present work has shown the feasibility of generating PDXs from breast cancer percutaneous needle biopsy samples in the neoadjuvant setting, both before and after chemotherapy. One advantage of our PDX models is that these PDXs were generated from patients who had extensive genomic and clinical follow-up information. The information from both patients and their xenografts will enable us to translate findings obtained from the PDX models to patient care.

## Conclusions

In summary, our study showed that PDX models developed from breast cancer percutaneous needle biopsy samples reliably represent human cancer biology. These pre-clinical models provide a unique opportunity to identify biomarkers and study mechanisms of treatment resistance to standard chemotherapy. They could also help future drug development by making it possible to test new antitumor agents or new combination therapy based on selective biomarkers.

## Additional files

Additional file 1: Figure S1. Characterization and utilization of PDX models generated from both pretreatment biopsies and surgical samples in the BEAUTY study. **Figure S2.** Representative immunohistochemistry shows the change of subtype from luminal B to triple negative. The histology is depicted using H&E staining and the expression of ER, PR, HER2, and Ki-67 is compared between the representative PDX (passage 2) and the corresponding human tumor (M06). **Figure S3.** Immunohistochemistry shows different subtypes for xenografts derived from the same original patient tumor. The representative PDX tumors at passage 2, and corresponding human tumor (M14) are shown. **Figure S4.** In vivo taxane response for the other six PDX models tested. Passage 4 tumors were used for the drug tests. (PDF 4901 kb)

Additional file 2: Supplementary methods. **Table S1.** Clinical parameters effects on pre-treatment biopsy PDX rate. **Table S2.** Clinical parameters effects on residual surgical PDX rate. **Table S3.** Patient clinical molecular subtype versus xenograft molecular subtype. **Table S4.** Xenograft pathological subtype changes recapitulated clinical subtype change in patients. **Table S5.** Patient pre-treatment pam50 subtype versus pam50 subtype. **Table S6.** Drug response in xenograft concordance with clinical drug response. (DOC 150 kb)

## Abbreviations

BC: Breast cancer
CR: Complete response
ER: Estrogen receptor
FISH: Fluorescence in situ hybridization
HER2: Human epidermal growth factor receptor 2
IHC: Immunohistochemical
NAC: Neoadjuvant chemotherapy
PDX: Patient-derived xenograft
PR: Progesterone receptor
PR: Partial response
PTB: Percutaneous tumor biopsies
SD: Stable disease
TN: Triple negative
