# Correction to: Establishing and characterizing patient-derived xenografts using pre-chemotherapy percutaneous biopsy and post-chemotherapy surgical samples from a prospective neoadjuvant breast cancer study

**DOI:** 10.1186/s13058-021-01420-z

**Published:** 2021-03-25

**Authors:** Jia Yu, Bo Qin, Ann M. Moyer, Jason P. Sinnwell, Kevin J. Thompson, John A. Copland, Laura A. Marlow, James L. Miller, Ping Yin, Bowen Gao, Katherine Minter-Dykhouse, Xiaojia Tang, Sarah A. McLaughlin, Alvaro Moreno-Aspitia, Anthony Schweitzer, Yan Lu, Jason Hubbard, Donald W. Northfelt, Richard J. Gray, Katie Hunt, Amy L. Conners, Vera J. Suman, Krishna R. Kalari, James N. Ingle, Zhenkun Lou, Daniel W. Visscher, Richard Weinshilboum, Judy C. Boughey, Matthew P. Goetz, Liewei Wang

**Affiliations:** 1 Department of Molecular Pharmacology and Experimental Therapeutics, Mayo Clinic, 200 First Street SW, Rochester, MN 55905 USA; 2 Department of Oncology, Mayo Clinic, Rochester, MN 55905 USA; 3 Department of Laboratory Medicine and Pathology, Mayo Clinic, Rochester, MN 55905 USA; 4 Department of Health Sciences Research, Mayo Clinic, Rochester, MN 55905 USA; 5 Department of Cancer Biology, Mayo Clinic, Jacksonville, FL 32224 USA; 6 Department of Surgery, Mayo Clinic, Jacksonville, FL 32224 USA; 7 Department of Hematology/Oncology, Mayo Clinic, Jacksonville, FL 32224 USA; 8 Affymetrix, now part of Thermo Fisher Scientific, Santa Clara, CA 95051 USA; 9 Department of Hematology/Oncology, Mayo Clinic, Scottsdale, AZ 85259 USA; 10 Department of Surgery, Mayo Clinic, Scottsdale, AZ 85259 USA; 11 Department of Radiology, Mayo Clinic, Rochester, MN 55905 USA; 12 Department of Surgery, Mayo Clinic, Rochester, MN 55905 USA; 13 Department of Surgery, Cedars-Sinai Medical Center, Los Angeles, CA 90048 USA

**Correction to: Breast Cancer Research 19, 130 (2017)**

**http://dx.doi.org/10.1186/s13058-017-0920-8**

Following publication of the original article [1], the authors would like to correct several typos in the article text:

1. Page 4-right panel, Line 10: “The take rate was 51.3% (21/39; 95% CI: 34.8-67.6%) in TN subtype;”. Take rate should be **20**/39 not 21/39. Percentages are correct.
2. Page 4-right panel, Line 21: The last 2 sentences were corrected to be consistent with Table 2 as follows: “There was no take among the 18 luminal tumors. However, five of nine TN, and one of the eight HER2+ post-chemotherapy residual tumors took (verified breast tumor).”
3. Page 4-right panel, Table 2: Total implanted for Luminal B should be **14** instead of 18. The total number of implanted 35 was correct.
4. Page 5-left panel, Line 15: HER2+ tumors (7/17 vs. 2/15) should be (7/**19** vs. 2/15). *P*-value is correct.
5. Page 5-left panel, Line 20, The number of luminal tumors should be 18 instead of 22.

The authors sincerely apologize for any inconvenience.
