# Supplementary material for: Establishing and characterizing patient-derived xenografts using pre-chemotherapy percutaneous biopsy and post-chemotherapy surgical samples from a prospective neoadjuvant breast cancer study
Source: Breast Cancer Res. 2017 Dec 6;19:130. doi: 10.1186/s13058-017-0920-8 (PMC5719923; doi:10.1186/s13058-017-0920-8)

Supplementary Figure S1

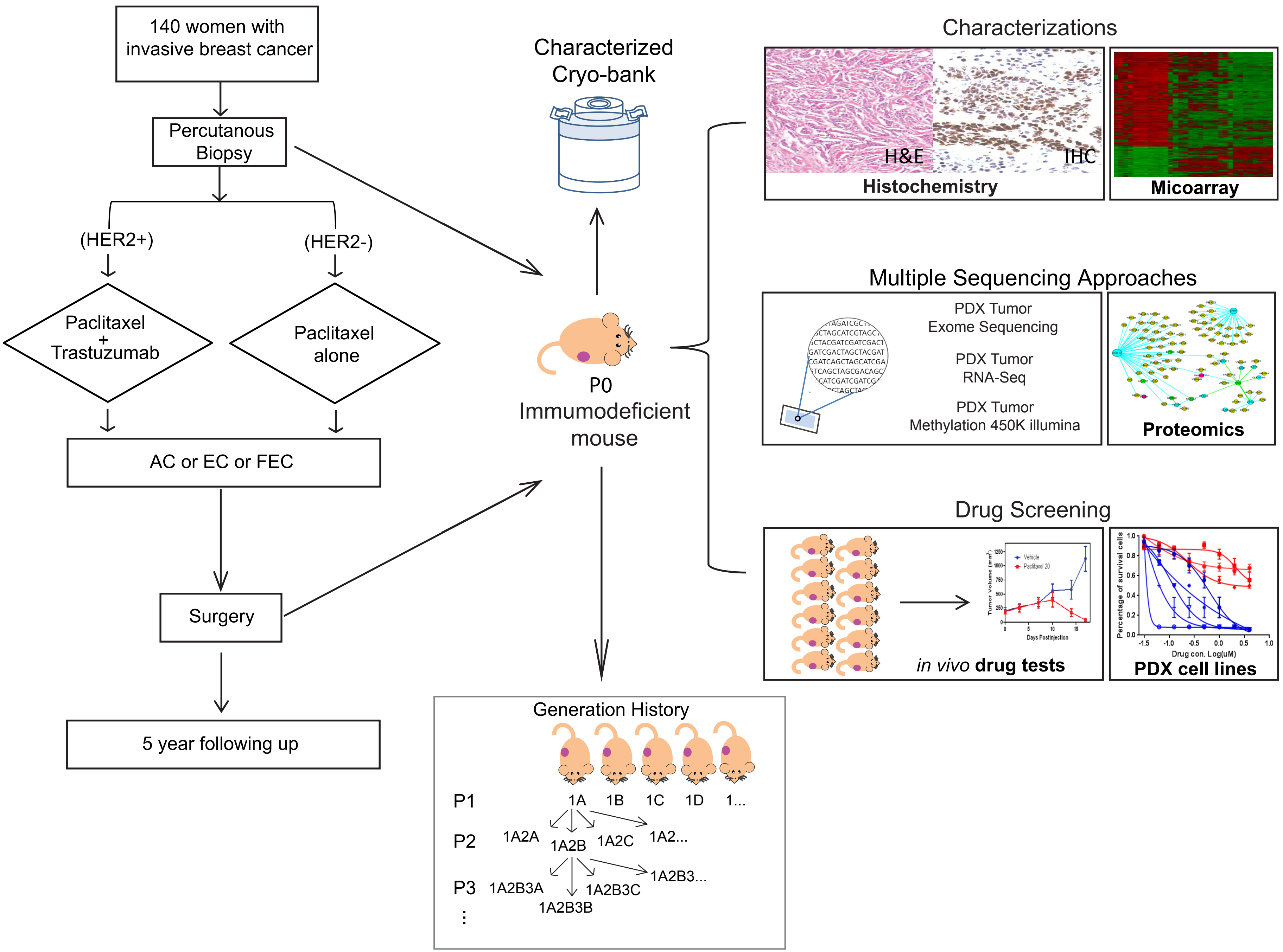

Supplementary Figure S2

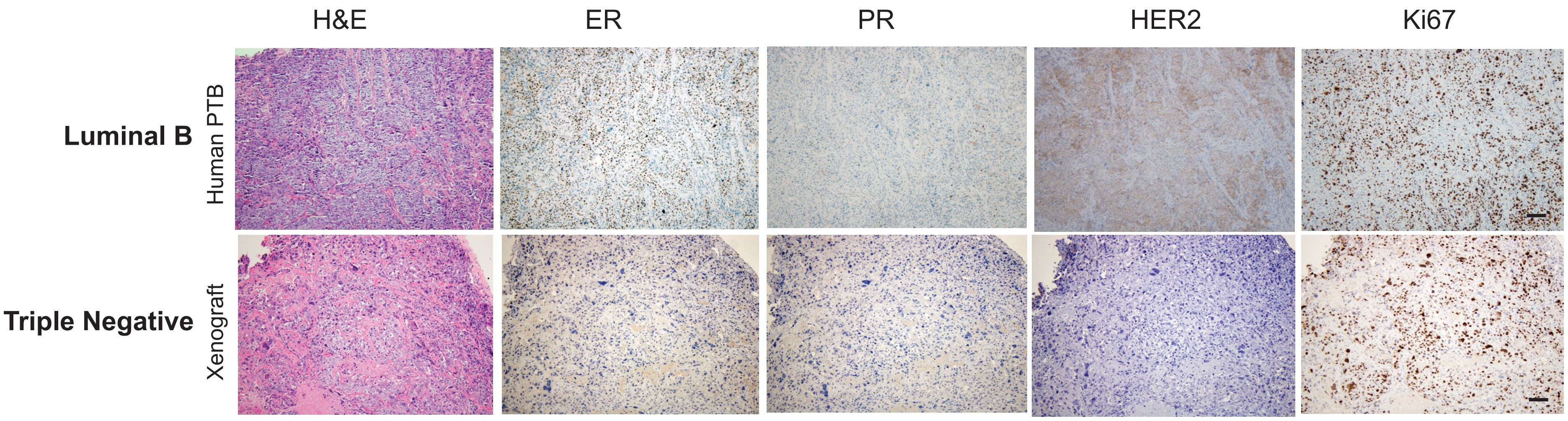

Supplementary Figure S3

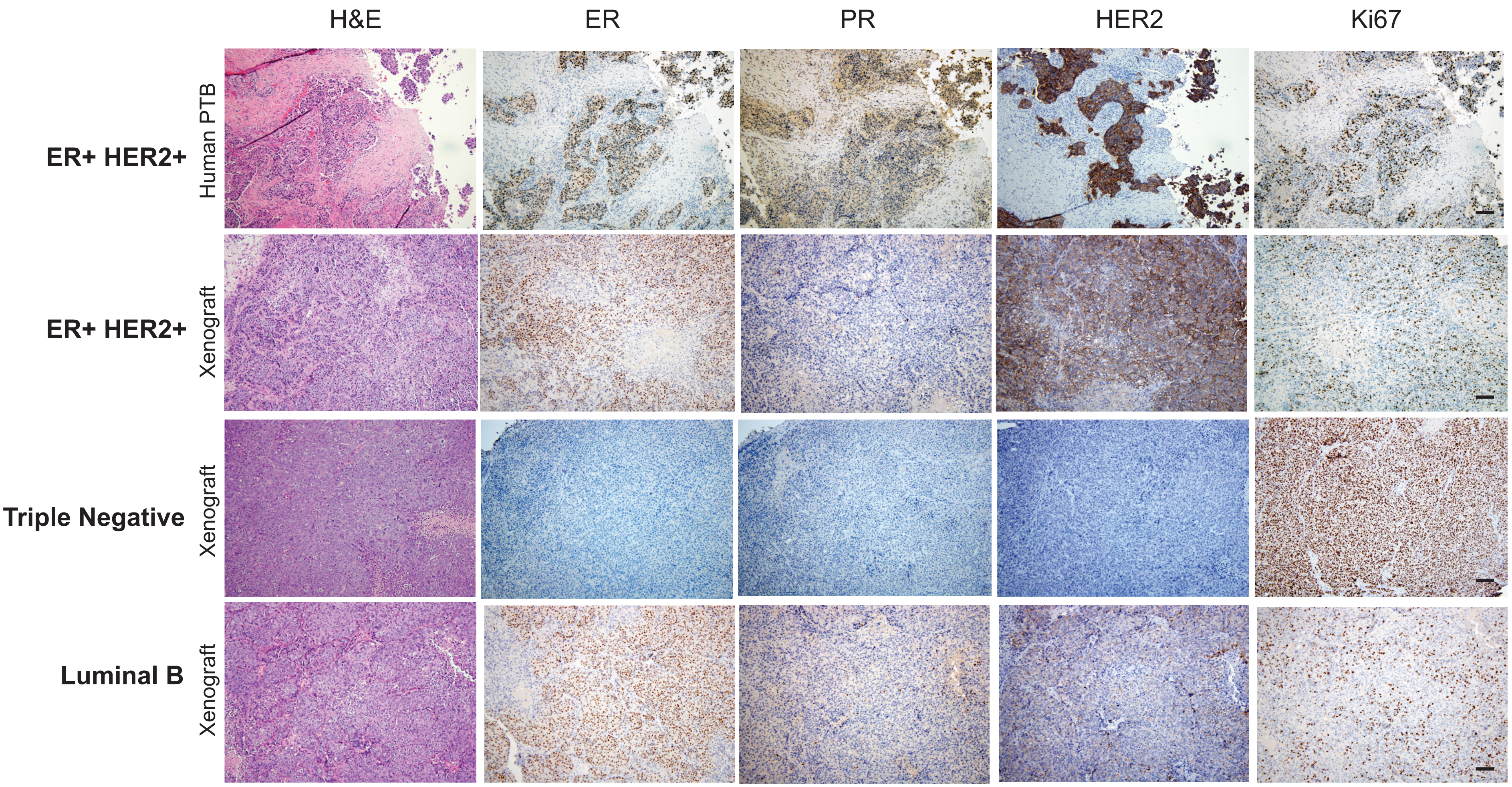

Supplementary Figure S4

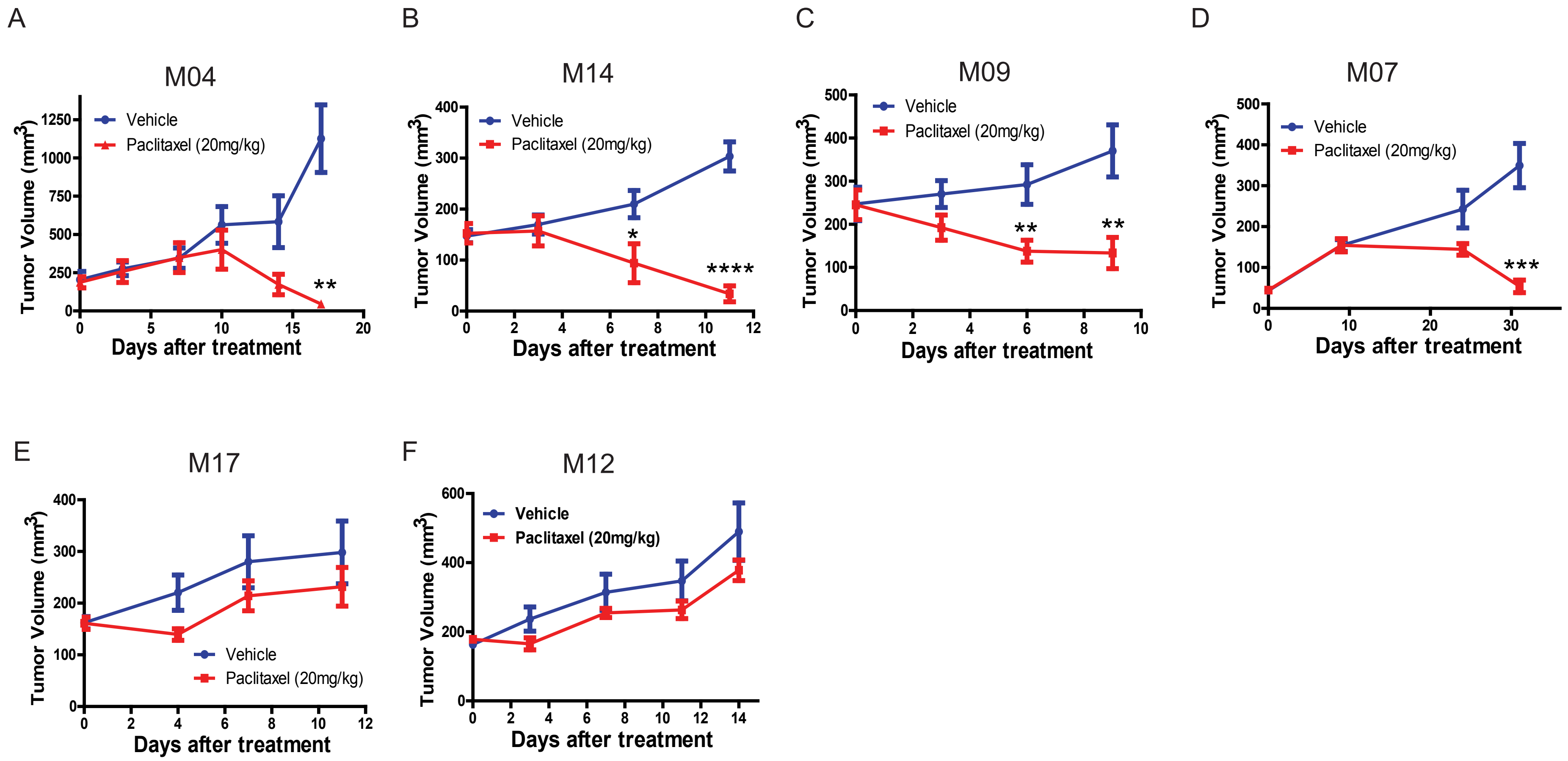

Supplement: Supplementary file 1 — Characterization and utilization of PDX models generated from both pretreatment biopsies and surgical samples in the BEAUTY study. Figure S2. Representative immunohistochemistry shows the change of subtype from luminal B to triple negative. The histology is depicted using H&E staining and the expression of ER, PR, HER2, and Ki-67 is compared between the representative PDX (passage 2) and the corresponding human tumor (M06). Figure S3. Immunohistochemistry shows different subtypes for xenografts derived from the same original patient tumor. The representative PDX tumors at passage 2, and corresponding human tumor (M14) are shown. Figure S4. In vivo taxane response for the other six PDX models tested. Passage 4 tumors were used for the drug tests. (PDF 4901 kb) [file 13058_2017_920_MOESM1_ESM.pdf]
