# Supplementary material for: Establishing and characterizing patient-derived xenografts using pre-chemotherapy percutaneous biopsy and post-chemotherapy surgical samples from a prospective neoadjuvant breast cancer study
Source: Breast Cancer Res. 2017 Dec 6;19:130. doi: 10.1186/s13058-017-0920-8 (PMC5719923; doi:10.1186/s13058-017-0920-8)
Supplement: Supplementary file 2 — Supplementary methods. Table S1. Clinical parameters effects on pre-treatment biopsy PDX rate. Table S2. Clinical parameters effects on residual surgical PDX rate. Table S3. Patient clinical molecular subtype versus xenograft molecular subtype. Table S4. Xenograft pathological subtype changes recapitulated clinical subtype change in patients. Table S5. Patient pre-treatment pam50 subtype versus pam50 subtype. Table S6. Drug response in xenograft concordance with clinical drug response. (DOC 150 kb) [file 13058_2017_920_MOESM2_ESM.doc]

**Establishing and characterizing patient-derived xenografts using pre-chemotherapy percutaneous biopsy and post-chemotherapy surgical samples from a prospective neoadjuvant breast cancer study**

Jia Yu^1^, Bo Qin^1,2^, Ann M. Moyer^3^, Jason P. Sinnwell^4^, Kevin J. Thompson^4^, John A. Copland III^5^, Laura A. Marlow^5^, James L. Miller^5^, Ping Yin^2^, Bowen Gao^13^, [Katherine Minter-Dykhouse](https://www.ncbi.nlm.nih.gov/pubmed/?term=Minter-Dykhouse K%5BAuthor%5D&cauthor=true&cauthor_uid=25732823)^2^, Xiaojia Tang^4^, Sarah A McLaughlin^6^, Alvaro Moreno-Aspitia^7^, Anthony Schweitzer^8^, Yan Lu^8^, Jason Hubbard^8^, Donald W. Northfelt^9^, Richard J. Gray^10^, Katie Hunt^11^, Amy L. Conners^11^, Vera J. Suman^4^, Krishna R. Kalari^4^, James N Ingle^2^, Zhenkun Lou^2^, Daniel W. Visscher^3^, Richard Weinshilboum^1^, Judy C. Boughey^12^, Matthew P. Goetz^1,2^, Liewei Wang^1#^

^1^Department of Molecular Pharmacology and Experimental Therapeutics; ^2^Department of Oncology; ^3^Department of Laboratory Medicine and Pathology; ^4^Department of Health Sciences Research, Mayo Clinic, Rochester, MN 55905, USA; ^5^Department of Cancer Biology; ^6^Department of Surgery; ^7^Department of Hematology/Oncology, Mayo Clinic, Jacksonville, FL 32224, USA; ^8^Affymetrix, now part of Thermo Fisher Scientific, Santa Clara, CA 95051, USA; ^9^Department of Hematology/Oncology; ^10^Department of Surgery, Mayo Clinic, Scottsdale, AZ 85259, USA; ^11^Department of Radiology; ^12^Department of Surgery, Mayo Clinic, Rochester, MN 55905, USA, ^13^Department of Surgery, Cedars-Sinai Medical Center, CA, 90048, USA

**^#^ Correspondence to:**

Liewei Wang, MD, PhD, Mayo Clinic, 200 First Street SW, Rochester, MN 55905, USA, email: [Wang.Liewei@mayo.edu](mailto:Wang.Liewei@mayo.edu), phone: 507-284-5264, fax 507-284-4455.

**Supplementary Methods**

**Mice maintenance and tumor implantation**

NOD-SCID (NOD.CB17-*Prkdc^scid^*/J) or NSG (NOD.Cg-*Prkdc^scid^ Il2rg^tm1Wjl^*/SzJ) mice were purchased from Jackson Laboratories (Bar Harbor, Maine) and kept under specific pathogen-free (SPF) conditions. Mice were pre-treated with 0.16µg/mL 17β-Estradiol in water at least one week before xenografting. 17β-Estradiol powder was purchased from Sigma Aldrich (St. Louis, MO). A stock solution of 8mg/ml 17β-Estradiol was prepared by dissolving the powder in 100% ethanol. This stock could be stored at -20 °C up to 3 months. Drinking water containing estradiol was made fresh every week.

Pre-neoadjuvant chemotherapy percutaneous biopsy specimen and post treatment surgical samples were delivered in sterile PBS and were received within one hour of the biopsy/surgery. Samples were then cut into 4 mm^3^ fragments and implanted subcutaneously with growth factor reduced Matrigel (purchased from BD Biosciences, Heidelberg, Germany) using a 14 gauge trocar. On average, 2 to 5 mice were implanted for each patient depending on the quality and quantity of samples received. Mice tumors were palpated weekly and tumor growth was measured using digital calipers. Tumor volumes were calculated using the formula π/6×L×W×H.

**Histological staining**

IHC was performed on human tumors using monoclonal antibodies against ER (SP1 clone, Ventana Medical Systems, Tucson, Arizona), PR (1E2 clone, Ventana Medical Systems), HER2 (FDA-approved Ventana Pathway HER2 using the 4B5 clone, Ventana Medical Systems), and Ki-67 (MIB-1 clone, Dako). For patient tumors, HER2 and Ki67 stains were scanned using the Aperio ScanScope instrument (Leica Biosystems, Richmond, Virginia) to capture digital images. A region including a minimum of 75% of the total invasive component of the carcinoma was identified by a trained technologist on each slide and included in the analysis for both HER2 and Ki67 using the Aperio ScanScope software. Membrane staining for HER2 was scored on a 0 to 3+ scale according to the CAP/ASCO guidelines. The percentage of cells staining positive for Ki-67 was analyzed. Scores for both HER2 and Ki67 staining were finalized by pathologist review. In the Pathology Research Core, IHC staining was performed on xenograft tumors using antibodies against ER (1D5 clone, Dako), PR (PgR 363 clone, Dako), HER2 (Herceptin Test kit, Dako), and Ki-67 (MIB-1 clone, Dako). ER and PR stains for both human and xenograft tumors were scored based on the following categories: negative (<1% reactive cells), focally positive (1-10% reactive cells), and positive (>10% reactive cells). For xenograft tumors, HER2 and Ki67 scoring was performed manually because of the cost associated with the use of the digital images for a large number of PDX tumors. HER2 scoring followed CAP/ASCO guidelines, while Ki67 was scored as <1%, 1-10%, 11-25%, 26-50%, 51-75%, 76-90%, and >90%. For the xenograft tumors that fell into the 11-25% Ki67 range, additional manual scoring was performed to establish whether the Ki67 score was ≤14% or >14% for comparison to the human tumor.

**Human Transcriptome Array HTA2.0 probes selection**

In our analysis of the microarray data, we had, on average ~130 probes per gene, from which we selected the best 10 probes representing the average gene expression level using the following criteria that were recommended by Affymetrix. Specifically, probes from the existing HTA-2_0 array were evaluated for 17mer hits against the mouse genome (mm10) and mouse transcriptome. That information was combined with existing information for the HTA-2_0 probes, which included 17mer hits to the human genome (hg19) and human transcriptome, as well as information regarding the composition of the probe itself (e.g. measurement of the probes’ GC composition, mononucleotide sequences, low complexity regions). For each criterion, a simplified score (0-9) was assigned, and a combined score was created by concatenating individual scores. Based on these scores, probes were assigned to tiers (Tier1 -> Tier3). Following probe scoring, probes were grouped by transcript cluster (TC) and then by probe selection region (PSR). Selection of the 10 best probes for a TC began by building a candidate probe pool. PSR probe pools were grouped by the percentage of transcripts (TRs) from the TC that overlapped with the PSR. In descending order, all probes from the highest available percent TR overlap with the PSRs group were added to the candidate probe pool until at least 10 probes were available. If probes from greater than 10 PSRs existed in the candidate probe pool, PSRs were sorted randomly and the highest Tier1 probe was selected from each PSR until 10 probes were selected. If 10 probes were not selected, the selection process continued with Tier2 and Tier3 probes. If probes from fewer than 10 PSRs were available, PSRs were shuffled, and the best probe from each PSR was selected until 10 probes have been selected.

**Xenograft mRNA extraction and qualification**

Total mRNA was isolated using the RNeasy Mini Kit (Qiagen). RNA quality was evaluated by measuring the RNA integrity number (RIN) using the Agilent RNA 6000 Nano or Pico assays. RIN number was determined using the Agilent 2100 Bioanalyzer (Agilent Technologies, Santa Clara, CA). Only samples with a RIN ≥6.0 were considered for further analysis.

**Gene expression analysis to compare xenograft similarity**

Unsupervised sample clustering was performed in Spearman’s correlation space (1-correlation) using complete linkage. The ape package was used to create a fanned dendrogram layout. Intra and inter sample correlations were computed for the 14 unique patient tumors represented by multiple xenografts, either from the same or different generations. Intrinsic modeling was performed using the PAM50 gene signature and was centered to 859 TCGA RSEM counts, and samples with Spearman’s correlations greater than 0.1 were classified to the maximally correlated subtype centroid . MAPT (microtubule associated protein Tau), a member of the intrinsic signature was measured by 2 probe sets on the HTA array. The two probe sets shared a correlation of 0.92 (Spearman’s) and were aggregated by their simple mean.

**Supplementary Tables**

**Table S1: Clinical Parameters Effects on Pre-Treatment Biopsy PDX Rate**

| Parameter | No PDX | PDX | Fisher’s exact test |
| --- | --- | --- | --- |
|  | (N=44) | (N=29) | p-value |
| Clinical Molecular Subtype | | | |
| HER2+ (n=34) | 25 (73.5) | 9 (26.5) | 0.0348 |
| Triple Negative (n=39) | 19 (48.7) | 20 (51.3) |  |
| Patient Age | | | |
| < 50 years old (n=44) | 27 (61.4) | 17 (38.6) | 0.9999 |
| ≥ 50 years old (n=29) | 17 (58.6) | 12 (41.4) |  |
| Clinical T-Stage | | | |
| 1-2 (n=36) | 21 (68.3) | 15 (41.7) | 0.8131 |
| 3-4 (n=37) | 23 (62.1) | 14 (37.9) |  |
| Clinical Nodal Stage | | | |
| N0 (n=33) | 20 (60.6) | 13 (39.4) | 1 |
| N1-N3 (n=40) | 24 (60.0) | 16 (40.0) |  |
| Mouse Strain | | | |
| NOD-SCID (n=47) | 35 (74.5) | 12 (25.5) | 0.0012 |
| NSG (n=26) | 9 (34.6) | 17 (65.4) |  |
| Nottingham Grade | | | |
| 1-2 (n=19) | 18 (94.7) | 1 (5.3) | 0.0003 |
| 3 (n=54) | 26 (48.1) | 28 (51.9) |  |
| Ki-67 | | | |
| ≤ 14  (n=5) | 3 (60.0) | 2 (40.0) | 0.9999 |
| > 14 (n=65) | 39 (60.0) | 26 (40.0) |  |

**Table S2: Clinical Parameters Effects on Residual Surgical PDX Rate**

| Parameter | No PDX  (N=11) | PDX  (N=6) | Fisher’s exact test  p-value |
| --- | --- | --- | --- |
| Clinical Molecular Subtype | | | |
| HER2+ (n=8)  Triple Negative (n=9) | 7 (87.5%)  4 (44.4%) | 1 (2.5%)  5 (55.6%) | 0.0882 |
| Residual Cancer Burden | | | |
| I-II (n=10)  III (n=7) | 6 (60.0%)  5 (71.4%) | 4 (40.0%)  2 (28.6%) | 0.8405 |
| Cellularity* | | | |
| ≤ 20% (n=7)  > 20% (n=10) | 6 (85.7%)  5 (50.0%) | 1 (14.3%)  5 (50.0%) | 0.1595 |
| Ki-67 | | | |
| ≤ 14 (n=5)  > 14 (n=9) | 5 (100%)  3 (33.3%) | 0  6 (66.7%) | 0.0280 |
| Mouse Strain | | | |
| NOD-SCID (n=15)  NSG (n=2) | 10 (66.7%)  1 (50.0%) | 5 (33.3%)  1 (50.0%) | 0.5956 |

* median of observed cellularity values observed

| **Table S3: Patient Clinical Molecular Subtype versus Xenograft Molecular Subtype** |
| --- |

| Patient Molecular Subtype* | HER2+ | LumA | LumB | LumUnk | Triple Negative |
| --- | --- | --- | --- | --- | --- |
| HER2+ | 8 | 0 | 1 | 0 | 0 |
| LumA | 0 | 0 | 0 | 0 | 0 |
| LumB | 0 | 0 | 1 | 0 | 1 |
| LumUnk | 0 | 0 | 0 | 0 | 0 |
| Triple Negative | 0 | 0 | 1 | 0 | 19 |

*The diagonal numbers include the total with at least one PDX was the same subtype as the patient sample, but the off-diagonal is the subtype of the majority of the PDXs generated from that patient sample that was not the same as the patient sample.

**Table S4. Xenograft Pathological Subtype Changes Recapitulated** Clinical Subtype Change in patients.

|  |  |  | **Base Line** | | | **Surgery** | | | |
| --- | --- | --- | --- | --- | --- | --- | --- | --- | --- |
| **Patient ID** | **Age** | **Race** | **Histology** | **Nottingham Grade** | **Clinical Molecular Subtype** | **surgery** | **Tumor laterality** | **Molecular Subtype (surgery)** | **Residual Cancer Burden Class** |
| M10 | 58 | White | Infiltrating ductal | 3 | ER+/HER2+ | yes | Left | Lum B | RCB-II |

| **Table S5: Patient Pre-treatment PAM50 Subtype versus PAM50 Subtype** |
| --- |

|  | Xenograft PAM50 Subtype | | | | |
| --- | --- | --- | --- | --- | --- |
| Patient PAM50 Subtype* | Basal | HER2 | Luminal A | Luminal B |  |
| Basal | 14 | 1 | 0 | 1 |  |
| HER2 | 0 | 4 | 0 | 1 |  |
| Normal | 0 | 1 | 0 | 0 |  |

*The diagonal numbers include the total with at least one PDX was the same subtype as the patient sample, but the off-diagonal is the subtype of the majority of the PDXs generated from that patient sample that was not the same as the patient sample.

**Table S6. Drug Response in xenograft concordance** with clinical drug response

|  | **Patients** | | | |  | **Xenografts** | | |
| --- | --- | --- | --- | --- | --- | --- | --- | --- |
| **ID** | **Therapy** | **Compound** | **Clinical Subtype** | **Clinical Response** |  |  | **Xenograft Response** | **Concordance** |
| **M04** | Neoadjuvant | Paclitaxel | TN | CR |  |  | Yes | Yes |
| **M01** | Neoadjuvant | Paclitaxel | TN | PR |  |  | Yes | Yes |
| **M13** | Neoadjuvant | Paclitaxel | TN | SD |  |  | No | Yes |
| **M07** | Neoadjuvant | Paclitaxel | TN | CR |  |  | Yes | Yes |
| **M17** | Neoadjuvant | Paclitaxel | TN | SD |  |  | No | Yes |
| **M14** | Neoadjuvant | Paclitaxel | ER+/HER2+ | PR |  |  | Yes | Yes |
| **M09** | Neoadjuvant | Paclitaxel | ER-/HER+ | PR |  |  | Yes | Yes |
| **M12** | Neoadjuvant | Paclitaxel | TN | SD |  |  | No | Yes |

**References:**

1. Levin-Allerhand JA, Sokol K, Smith JD: **Safe and effective method for chronic 17beta-estradiol administration to mice**. *Contemp Top Lab Anim Sci* 2003, **42**(6):33-35.

2. Pearse G, Frith J, Randall KJ, Klinowska T: **Urinary retention and cystitis associated with subcutaneous estradiol pellets in female nude mice**. *Toxicol Pathol* 2009, **37**(2):227-234.

3. Paradis E, Claude J, Strimmer K: **APE: Analyses of Phylogenetics and Evolution in R language**. *Bioinformatics* 2004, **20**(2):289-290.

4. Mackay I, Horwell A, Garner J, White J, McKee J, Philpott H: **Reanalyses of the historical series of UK variety trials to quantify the contributions of genetic and environmental factors to trends and variability in yield over time**. *Theor Appl Genet* 2011, **122**(1):225-238.
